# Acute posterior neck pain in adult: a case series

**DOI:** 10.4076/1757-1626-2-8103

**Published:** 2009-08-25

**Authors:** Yasuhiro Homma, Akira Itoi, Tomoya Muta, Yoshio Shimamura, Kiyohito Naito, Atsuhiko Mogami, Osamu Obayashi, Colin G Murphy, Kazuo Kaneko

**Affiliations:** Department of Orthopaedic Surgery, Juntendo University Shizuoka HospitalIzunokuni City, Shizuoka 410-2295Japan

## Abstract

Calcification around the cervical spine is thought to be relatively rare case, among these conditions the calcification at the longus colli muscle is called retropharyngeal tendinitis and only several cases were reported in the literature. In this disease, the three characteristic clinical features are acute severe posterior neck pain without any trigger, severely restricted range of motion, and odynophagia. The radiographic findings include swelling of the retropharyngeal space and amorphous calcification anterior to C1-C2 in lateral view of the cervical spine. Diagnosis is established by sound history taking coupled with computed tomography (CT) scan of the affected area, treatment is the administration of oral non-steroidal anti-inflammatory drugs (NSAID), for a few weeks and/or oral steroid. We present a new 3 cases (27, 35, and 24 years old, male) and review of literature.

## Introduction

Calcifications around the shoulder, the elbow, and the hip are common, can cause severe pain, and restrict range of motion. Although described [1,2,3], calcification around the cervical spine is less well known, but can cause significant problems. Here, we present three patients with retropharyngeal calcific tendinitis and review of literature.

## Case presentation

### Case report 1

**Figure 1.**
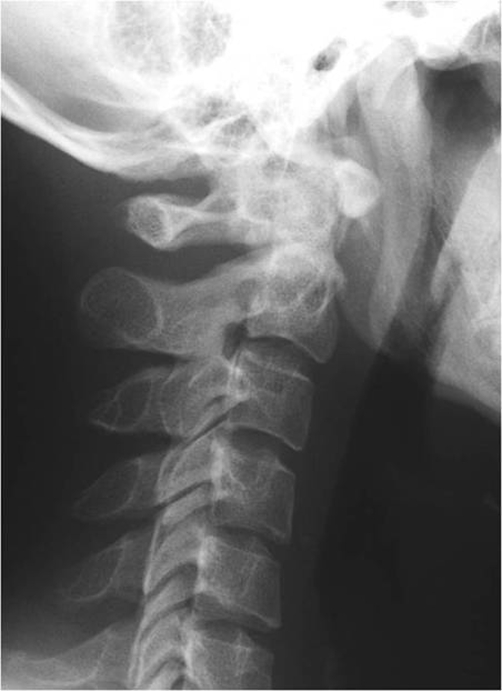
Retropharyngeal thickening and soft tissue calcification in front of the anterior arch of C1.

**Figure 2.**
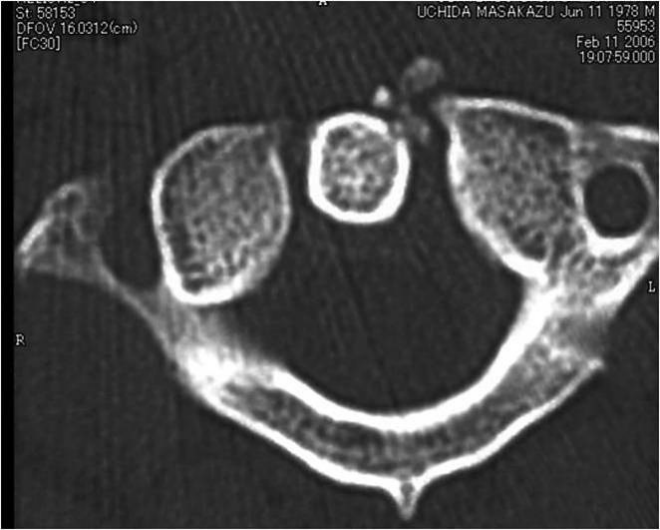
Soft tissue calcification from the longus colli muscle to the atlanto-odontoid joint.

**Figure 3.**
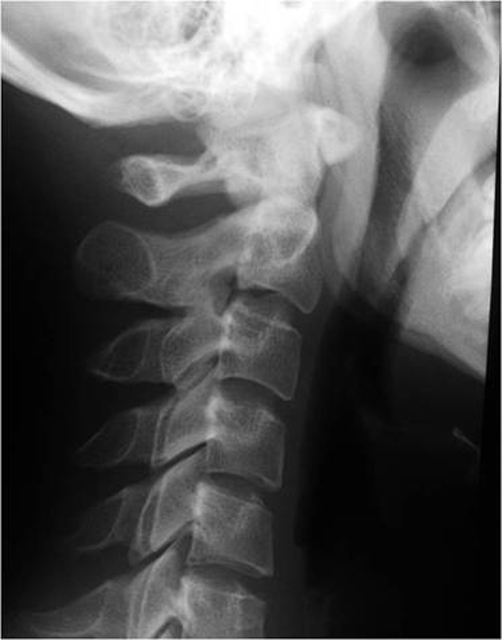
No swelling of retropharyngeal space.

**Figure 4.**
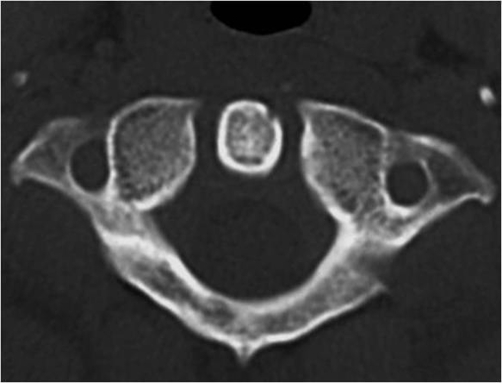
No soft tissue calcification in an unenhanced CT of cervical spine.

Patient A is a 27-year-old Japanese male who presented with a 2-day history of a posterior neck pain, sore throat, and odynophagia. There was no history of trauma, upper respiratory infection, or dental problems. On examination, active range of cervical motion was severely reduced by pain, localized to the posterior neck. There was no abnormal neurological finding. Oropharyngeal examination was normal. His temperature was 37.5°C. White blood-cell (WBC) count was 12,500/mm^3^, C-reactive protein (CRP) was 1.0 mg/dl. Lateral view of cervical X-ray revealed retropharyngeal thickening at the upper cervical spine and soft tissue calcification in front of the anterior arch of C1 (Figure 1). An unenhanced CT demonstrated the soft tissue calcification from the longus colli muscle to the atlanto-odontoid joint (Figure 2). Antibiotic and NSAID were administered, because infection of the pharynx was suspected. The next day, oral prednisone was added because there was no change of the pain. The symptoms resolved shortly thereafter. On day 4 post initial examination, the patient’s cervical pain had improved, active range of motion was almost normal, there was no odynophagia. WBC and CRP were decreased. On serial reviews at days 11 and 26 following first examination, the patient was asymptomatic with no recrudescence. Interval X ray showed no swelling of retropharyngeal space (Figure 3). There was no soft tissue calcification in an unenhanced CT of cervical spine (Figure 4).

### Case report 2

**Figure 5.**
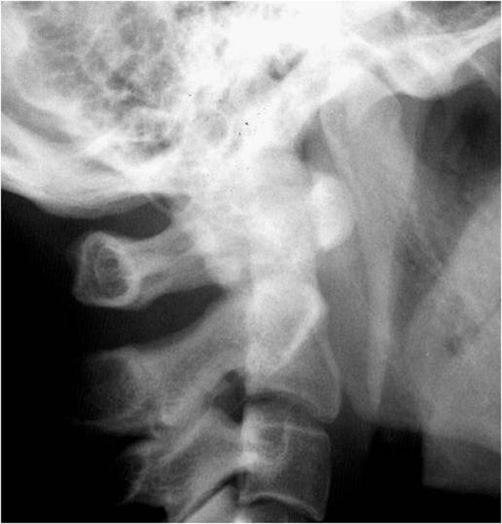
Swelling of the retropharyngeal space.

**Figure 6.**
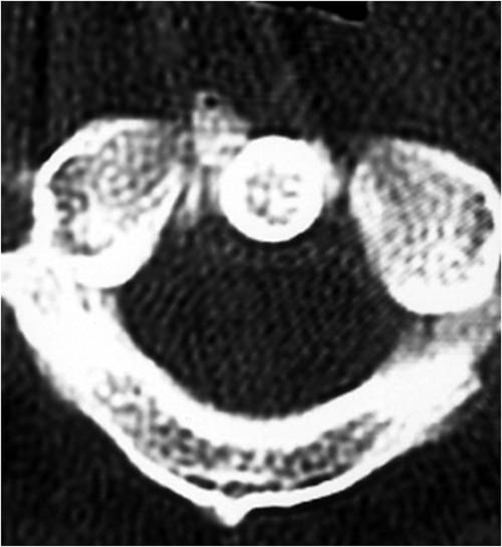
The calcification anterior to the dens.

Patient B is a previously healthy 35-year-old Japanese male who awoke three days prior to presentation with severe posterior neck pain. On examination, neck movement was restricted due to severe pain. There was no neurological deficit. WBC and CRP were mildly raised. Lateral plain film of the cervical spine revealed swelling of the retropharyngeal space (Figure 5). CT scan of the C-spine showed the calcification anterior to the dens (Figure 6). Symptoms resolved quickly with the administration of oral NSAID.

### Case report 3

**Figure 7.**
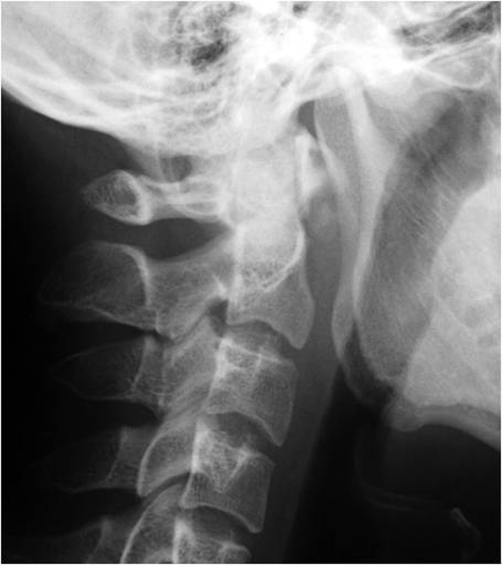
Swelling of the retropharyngeal space and calcification anterior to the dens.

**Figure 8.**
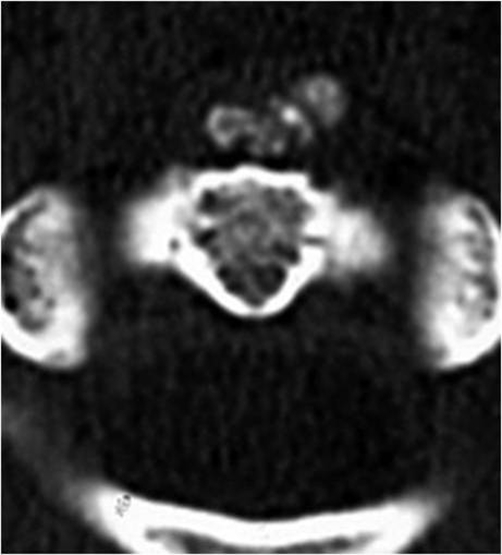
Swelling of the retropharyngeal space and calcification anterior to the dens.

Patient C is a 24-year-old otherwise healthy Japanese male who presented with a 6 day history neck pain. The pain increased in severity until his presentation to the outpatient clinic. On examination there was a severely limited range of motion of the neck due to posterior neck pain. No neurological finding was detected. X-ray and CT scan showed swelling of the retropharyngeal space and calcification anterior to the dens (Figures 7,8). After administration of NSAID for seven days, the symptoms resolved fully.

## Discussion

Calcification around the cervical spine has been thought to be very rare condition and its pathology has been unclear. The calcium deposit at the longus colli muscle is called retropharyngeal calcific tendinitis, also called calcific prevertebral tendinitis, calcific tendinitis of the longus colli muscle. Since Hartley first described this condition in 1964 [1], there have some case reports on the literature [2-5]. It remains under-diagnosed however, possibly through lack of recognition or awareness.

The condition occurs most frequently in the third through sixth decade of life, although it has been reported in adults between the ages of 21 and 81 years. The pathology is not entirely clear, with one report by Ring et al. suggesting that examination under light microscopy of the tissue sections revealed intratendinous calcium granuloma lesions [5] with fragments of skeletal muscle and fibrous tissue [6].

The three clinical features of this condition are acute severe posterior neck pain without any trigger, severely restricted range of motion, and odynophagia. Mild headache may sometimes be reported. These symptoms may be associated with low grade fever. Laboratory data shows mild elevation of WBC and CRP. The radiographic findings include swelling of the retropharyngeal space and amorphous calcification anterior to C1-C2 in lateral view of the cervical spine. CT scan is very useful in identifying the calcification, in addition to out ruling other pathologies such as fracture or abscess. Although judging from CT scan of case A, this calcification can be transient, like the calcification in other joints, its pathophysiology is not clear yet.

The differential diagnosis would include traumatic fracture-dislocation of the vertebra, retropharyngeal abscess, meningitis, and infectious spondylitis.

Treatment is the administration of oral NSAID for 1 to 2 weeks and/or oral steroid. The process is thought to be self-limiting [7]. When infection is suspected, enhanced CT scan and laboratory test are necessary to rule out an abscess or infectious spondylitis.

As in our three cases, all patients have a similar medical history, and self limiting clinical course. Although majority of patients presenting with this condition will be seen and treated by primary health care physicians, orthopaedic referrals are not unusual for atypical neck pain. In this context we feel these cases show the importance of awareness of the condition, and highlight the necessity for sound history taking and appropriate laboratory and radiographic investigations to avoid unnecessary further investigations or treatment.

## Abbreviations

CT: computed tomography
CRP: C-reactive protein
NSAID: non-steroidal anti-inflammatory drugs
WBC: white blood cell
